# Quorum sensing modulates microbial community structure through regulation of secondary metabolites

**DOI:** 10.1128/msphere.01050-24

**Published:** 2025-06-20

**Authors:** April C. Armes, Amy L. Schaefer, Leah H. Hochanadel, Dawn M. Klingeman, Dana L. Carper, Paul E. Abraham, Larry M. York, Alyssa A. Carrell, Mitchel J. Doktycz, Dale A. Pelletier

**Affiliations:** 1 Biosciences Division, Oak Ridge National Laboratory, Oak Ridge, Tennessee, USA; 2 University of Washington 7284, Seattle, Washington, USA; The University of Texas Medical Branch at Galveston, Galveston, Texas, USA

**Keywords:** AHLs, quorum sensing, quorum quenching, SynComs, phenazine, polymicrobial, RAMs, microbial communities, lactonase

## Abstract

Bacteria are recognized for their diverse metabolic capabilities, yet the impact of microbe-microbe interactions on multispecies community structure and dynamics is poorly understood. Cell-to-cell signaling in the form of quorum sensing (QS) often regulates secondary metabolite production and microbial interactions. Here, we examine how acylhomoserine lactone (AHL)-mediated QS impacts microbial community structure in a 10-member synthetic community of isolates from *Populus deltoides*. To explore the role of QS in microbial community structure and dynamics, we disrupted AHL signaling by exogenous addition of AiiA-lactonase, an enzyme that cleaves the lactone ring. Microbial community structure resulting from signal inactivation, as measured by 16S rRNA amplicon sequencing and secondary metabolite production, was assessed after successive passaging of the community. Further, we investigated the impact of quorum quenching on specific microbe-microbe interactions using pairwise inhibition assays. Our results indicate that AHL inactivation alters the relative abundance of dominant community members at later passages but does not impact the overall membership in the community. Quorum quenching significantly alters the metabolic profile in lactonase-treated communities. This metabolic alteration impacts microbe-microbe interactions through decreased inhibition of other community members. Together, these results indicate that QS impacts microbial community structure through the regulation of secondary metabolites in dominant members and that the membership of microbial communities can be relatively stable despite changes in metabolic profiles.

**IMPORTANCE:**

In terrestrial ecosystems, bacteria exist as multispecies consortia and provide diverse ecosystem services. Interactions among microbes contribute to determining their abundance and population structure and are often mediated by cell-to-cell communication. However, the role of microbial communication in community assembly is poorly understood. In this study, we investigated the disruption of AHL-based quorum sensing on bacterial community structure using a synthetic microbial community derived from a plant host. We found that disrupting AHL signaling did not change the membership but shifted the relative abundance of the dominant community members. Metabolic profiles of disrupted communities reveal alterations in key secondary metabolites that likely reduce antagonistic behavior. Investigating the driving mechanisms underlying microbial community assembly is fundamental to understanding microbial ecosystem ecology and can be broadly applied toward understanding sustainable systems and facilitating agricultural applications where plant-associated microbes are of growing importance.

## INTRODUCTION

Microbial communities are vital to many ecosystem processes. In terrestrial systems, diverse microbial communities drive a range of processes: from decomposing organic matter and cycling nutrients in soil to fixing nitrogen and aiding plant health through enhanced nutrient uptake and resistance to pathogens (1). Trophic interactions and signal exchange between microorganisms within these communities influence community structure (i.e., relative abundance of community members) and function (i.e., what they are doing). Many of these dynamic interactions are mediated by a regulatory mechanism known as quorum sensing (QS) (2, 3).

Quorum sensing in bacteria regulates essential processes such as monitoring environmental conditions and regulating social behaviors involved in bacterial survival (4). QS depends on the synthesis of small diffusible signal molecules known as autoinducers (AI). Local concentrations of AI elicit a quorum by binding to their cognate transcriptional regulators. Once bound, population-level behaviors are facilitated. A prevalent class of AIs among Proteobacteria are acylhomoserine lactones (AHLs). AHL-mediated quorum sensing typically comprises an AHL synthase (*luxI* homolog) and a transcriptional regulator (*luxR* homolog) (5). AHLs are composed of a lactone ring and acyl carbon chain (6). Alterations in the length and saturation level of the acyl chain influence the binding affinity to the *luxR* homologs (7), which in turn impacts the genetic response (4, 8, 9). Through the production, release, and detection of AHLs, QS influences metabolic activity integral to the functionality and stability of a microbial community (10).

Despite the role QS plays in regulating population-level behaviors in bacteria, few efforts have been made to characterize its role in microbial communities comprising diverse organisms. Many studies have focused on examining the role of QS between bacteria in co-culture (11–17). The spatial dynamics of QS have been investigated using heterogeneous populations of single bacterial strains, demonstrating signal propagation through communities (18–20). More recently, studies have begun to examine QS using polymicrobial synthetic communities (SynComs) to better understand the role of QS in governing microbial community structure and dynamics (21–23). SynComs provide the advantage of reducing community complexity compared to natural environments while maintaining higher-order level interactions, making them ideal model systems to investigate the role of QS on microbial community structure and dynamics.

**Fig 1:**
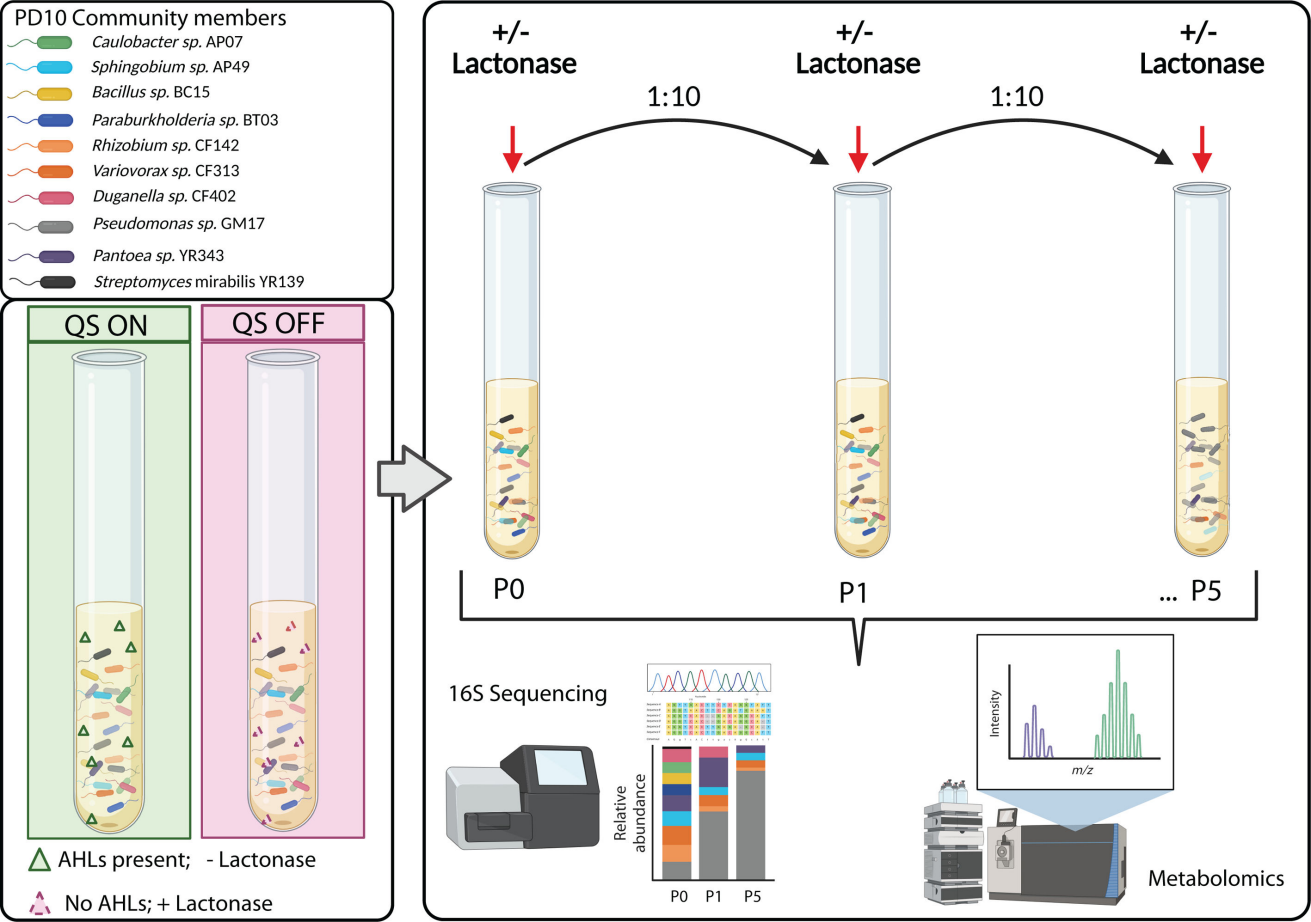
Overview of synthetic community and serial passage experimental design. Illustration created with Biorender.com.

In previous work, we utilized a 10-member SynCom, designated PD10, and demonstrated that this microbial community stabilized over time in passaging experiments to a defined subset of organisms. Further, the final community structure was unchanged despite significant differences in initial inoculum ratios (24, 25). PD10 consists of representative genome-sequenced isolates primarily composed of Proteobacteria from the *Populus deltoides* rhizosphere (24, 26–28). AHL synthase and receptor genes are prevalent among our *Populus* isolates (29), and given the global regulatory role of QS and its demonstrated importance in microbial communities (30), we hypothesized that AHL signal exchange contributes to microbial community structure and dynamics. To test this hypothesis, we disrupted AHL accumulation in PD10 cultures using purified AiiA-lactonase (lactonase hereafter), an enzyme that cleaves the lactone ring (Fig. 1). This allows a facile approach to manipulating QS in synthetic communities. Here, we evaluated the impact of AHL QS on microbial community structure and dynamics by 16S rRNA amplicon sequencing, metabolomics, and pairwise interaction screens.

## RESULTS

Strains selected for the PD10 community were originally isolated from the *Populus* rhizosphere and are representative of the major classes found in the *Populus* microbiome (26–28, 31). We examined the genomes of our PD10 SynCom isolates and identified AHL synthase and receptor genes in six of the ten isolates and found AHL signal determination for many of these strains (Table 1). To investigate the contribution of AHL-based QS in our SynComs, we exogenously added lactonase to one set of PD10 SynComs passaged in MOPS minimal medium with glucose as the sole carbon source. We first confirmed the absence of AHL activity in lactonase-treated cultures using an *Agrobacterium tumefaciens* reporter, which detects a wide range of AHL signals (32). Our data indicated that lactonase was able to suppress AHL production in PD10 cultures for 48 h (Fig. S1). With the confirmation that lactonase was active in our culture conditions, we moved forward with experiments to assess the impact of AHL QS on community structure.

**TABLE 1:** Table of LuxIR genes in PD10 community members^*d*^

Strain	# LuxR/I homologs	Lux I locus ID	LuxR locus ID	AHL activity^*a*^	AHL product^*b*^
*Caulobacter* sp. AP07	1	PMI01_00487	PMI01_02945	ND	Undefined
*Sphingobium* sp. AP49	1	PMI04_04262	PMI04_04261	Yes	Undefined
*Bacillus* sp. BC15	0	NA	NA	NA	NA
*Paraburkholderia* sp. BT03	1	PMI06_05109	PMI06_05111	Yes	3oxoC10^*b*^
*Rhizobium* sp. CF142	2	PMI11_05427; PMI11_05758	PMI11_05428; PMI11_05757	Yes	3OHC14:1^*b*^; C6^*b*^
*Variovorax* sp. CF313	0	NA	NA	NA	NA
*Duganella* sp. CF402	0	NA	NA	NA	NA
*Pseudomonas* sp. GM17	2	PMI20_01270; PMI20_01530;	PMI20_01269; PMI20_01529	Yes	3OHC6^*c*^; C4^*c*^
*Streptomyces mirabilis* YR139	0	NA	NA	NA	NA
*Pantoea* sp. YR343	1	PMI39_00508	PMI39_00509	ND	Undefined

^*a*^ (29).

^*b*^ Unpublished data.

^*c*^ (33).

^*d*^ NA—not applicable; ND—not detected.

### Quorum quenching alters community structure

**Fig 2:**
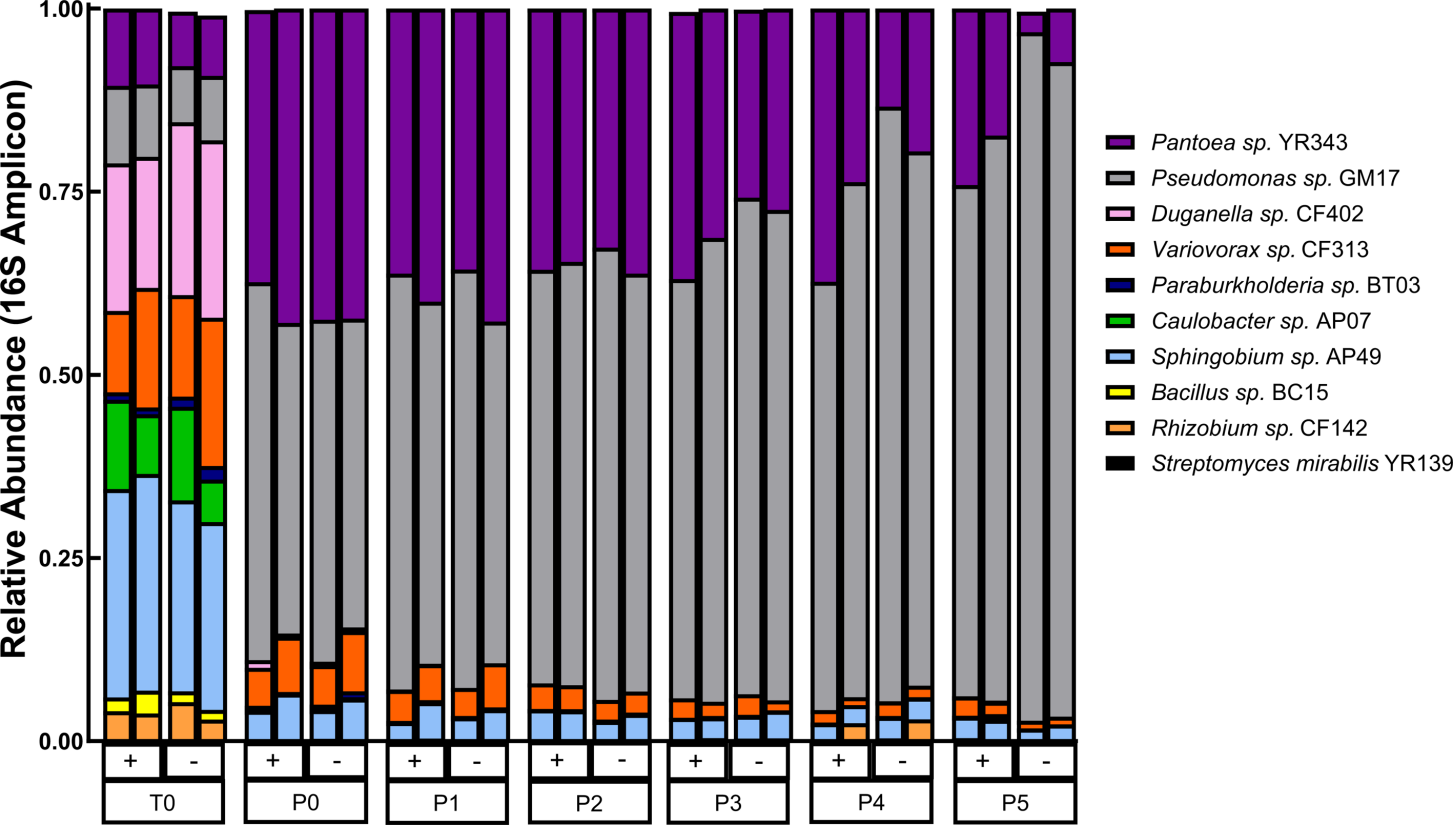
Community composition (16S) of PD10 SynCom treated with (+) and without (–) lactonase at each passage. Every stacked bar is the average of three technical replicates. Biological replicates from two independent experiments are in side-by-side bar plots. The *X*-axis represents the time of each passage, which occurred every 48 h. T0 is the initial seeding inoculum, followed by passage 0 (P0), passage 1 (P1), passage 2 (P2), passage 3 (P3), passage 4 (P4), and passage 5 (P5). Community members are denoted by color, as indicated by the legend.

To investigate the role of quorum quenching (QQ) in microbial community structure, we serially passaged mixed cultures with and without lactonase every 48 h in a 1:10 dilution in MOPS minimal media for a total of five passages. In the two independent passage experiments conducted, we found modest differences in the community structure between the two treatments, despite observing no statistical differences in growth parameters between treatments (Fig. S2). The taxonomic composition of the synthetic communities revealed four dominant strains at the end of five passages: *Pseudomonas* sp. GM17, *Pantoea* sp. YR343, *Sphingobium* sp. AP49, and *Variovorax* sp. CF313 accounted for 92%, 5%, 2%, and 1% of total 16S amplicon sequences, respectively, in cultures without lactonase (– lactonase, Table S2). Strain membership was similar regardless of whether the community was treated with lactonase. For example, GM17, YR343, AP49, and CF313 were all present in lactonase-containing cultures after five passages (p5). However, treatment with lactonase resulted in a significant shift in relative abundances of these members (GM17 = 73% [*P* = 0.004]; YR343 = 21% [*P* = 0.00005]; AP49 = 3% [*P* = 0.032]; CF313 = 2% [*P* = 0.015]) compared to lactonase-free cultures. Particularly, the relative abundance of YR343 and GM17 was drastically altered at later passages (Fig. S3). By the end of the experiment, the relative abundance of GM17 decreased by nearly 20% with lactonase addition, while relative abundances of YR343 (~16%), and to a lesser extent, AP49 (~1%) and CF313 (~1%), increased when AHL QS was suppressed (Fig. 2). These results are similar to previously published data, where GM17 dominated MOPS community structure (24).

To assess treatment effects on community structure diversity, we conducted beta diversity analysis using Bray-Curtis dissimilarity and performed permutational multivariate analysis of variance (PERMANOVA) to calculate statistical significance between community compositions across passages and treatment (Fig. S4). While initial community composition was not significantly different between treatments, treatment-specific differences became more defined with each passage (*P* < 0.05, *R*^2^ = 0.918) (Fig. S4). Over successive passages, community structures diverged significantly with increasing passage number and treatment, which independently explained 73.3% and 26.3% of the variance, respectively (*P* < 0.05, *R*^2^ = 0.014) (Table S3). Moreover, the interaction between passage, treatment, and experiment showed a substantial impact on community composition, explaining a combined 93.7% of the variance (Table S5). Specifically, at later passages (i.e., p4 and p5), treatment with lactonase had a more pronounced impact on community structure as communities became significantly and increasingly dissimilar (*R*² =0.958, *P* = 0.001). Across all passages, community composition varied significantly with the interaction of passage and treatment, suggesting that response to treatment is dependent on passage number (*P* = 0.001) (Table S4).

### Metabolites are differentially abundant in the presence of lactonase

We utilized LC-based metabolomics to assess metabolite composition between lactonase-treated and untreated cultures. We performed metabolite identification at Class 2 confidence levels, which signifies a probable assignment based on spectral matching with reference spectral library data. This level of identification allows for reliable metabolite characterization across large data sets and enables robust comparisons across studies (34). In total, 1,430 putative metabolites were identified through spectral matching against the 2020 High-Resolution NIST library and the GNPS public spectral library (Table S6). Clustering of metabolomic data based on treatment was followed by passaging to highlight distinct metabolite profiles between treatments and over time (Fig. S5). Furthermore, non-metric multidimensional scaling (NMDS) analysis showed a clear separation among groups, suggesting both treatment and passage number contribute to variations in metabolite composition (Fig. S6).

To determine the effects of treatment and passage on metabolites in the PD10 SynComs, we performed PERMANOVA. Regardless of treatment, metabolic profiles were significantly different across passages (*P* = 0.001). In fact, 38.6% of variation in data can be explained by the effect of passaging the community, consistent with the differences in community member growth over time (Fig. 2). The interaction between passage and treatment conditions also significantly contributed to the observed variations (*P* = 0.001), explaining 14.1% of the total variation, as evidenced by shifts in clustering patterns across different passages and treatments (Table S5; Fig. S6). Finally, treatment effects on community metabolic profiles were also significant (*P* = 0.006, *R*^2^ = 0.037) and coincide with the treatment effects seen in our community composition data (Table S3; Fig. S4). Together, these results indicate that passage and the combination of passage and lactonase treatment have a larger effect on community metabolic profiles than treatment alone.

**Fig 3:**
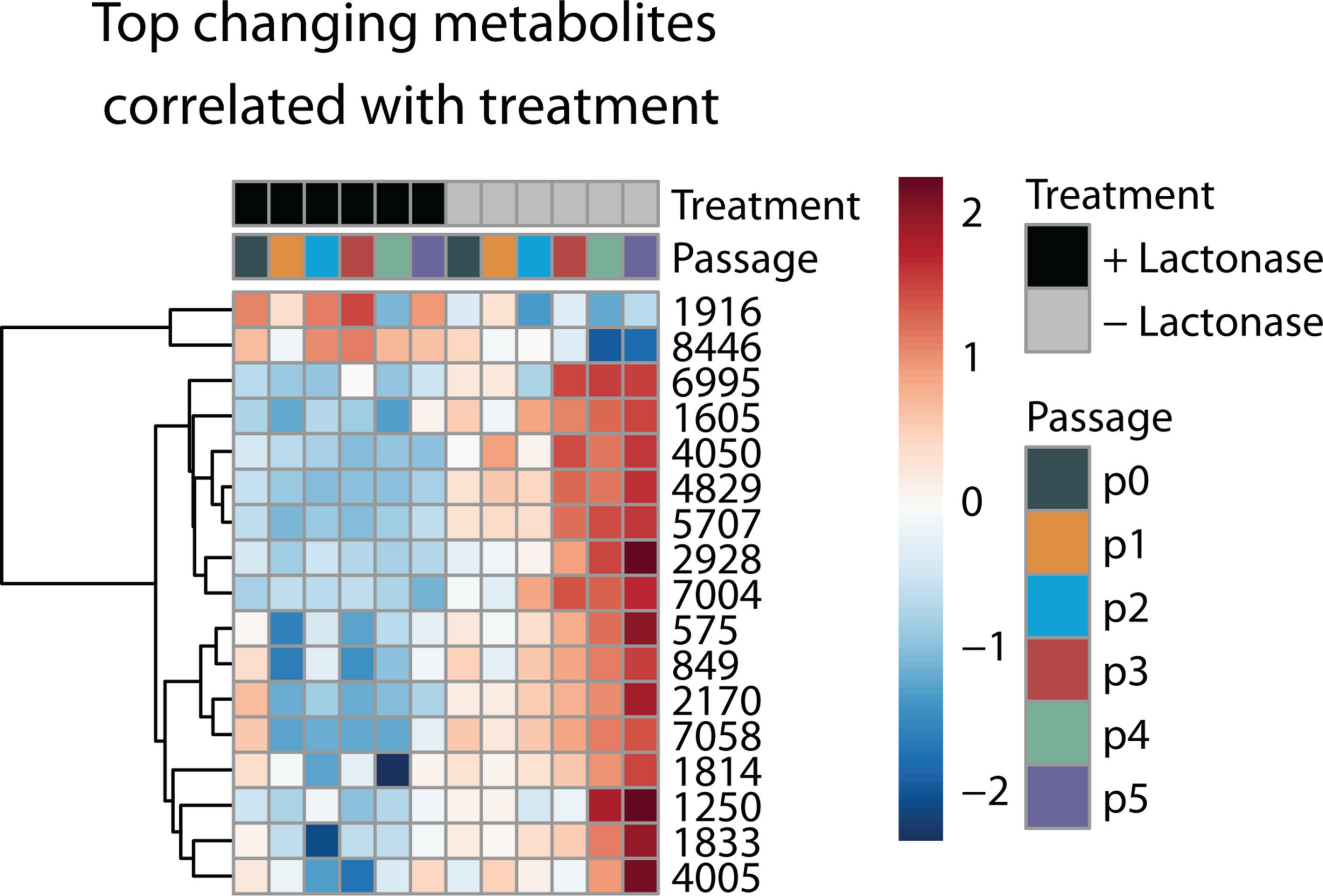
Heatmap of top changing metabolites correlated with lactonase treatment using a 0.5 correlation coefficient cutoff. The metabolites are selected based on their significant changes in response to treatments with (black) and without lactonase (gray). Rows represent individual metabolites as indicated by compound number designation, and columns represent different passages and treatments. Positive (red) and negative (blue) correlations are indicated by the color scale. Pearson’s correlation was used to quantify the degree of association between the metabolites and the respective treatments. Compounds 1,250, 1,605, 1,814, and 1,833 were identified as C6-HSL, 3OHC6-HSL, phenazine-1-carboxamide, and phenazine-1-carboxylic acid, respectively.

**Fig 4:**
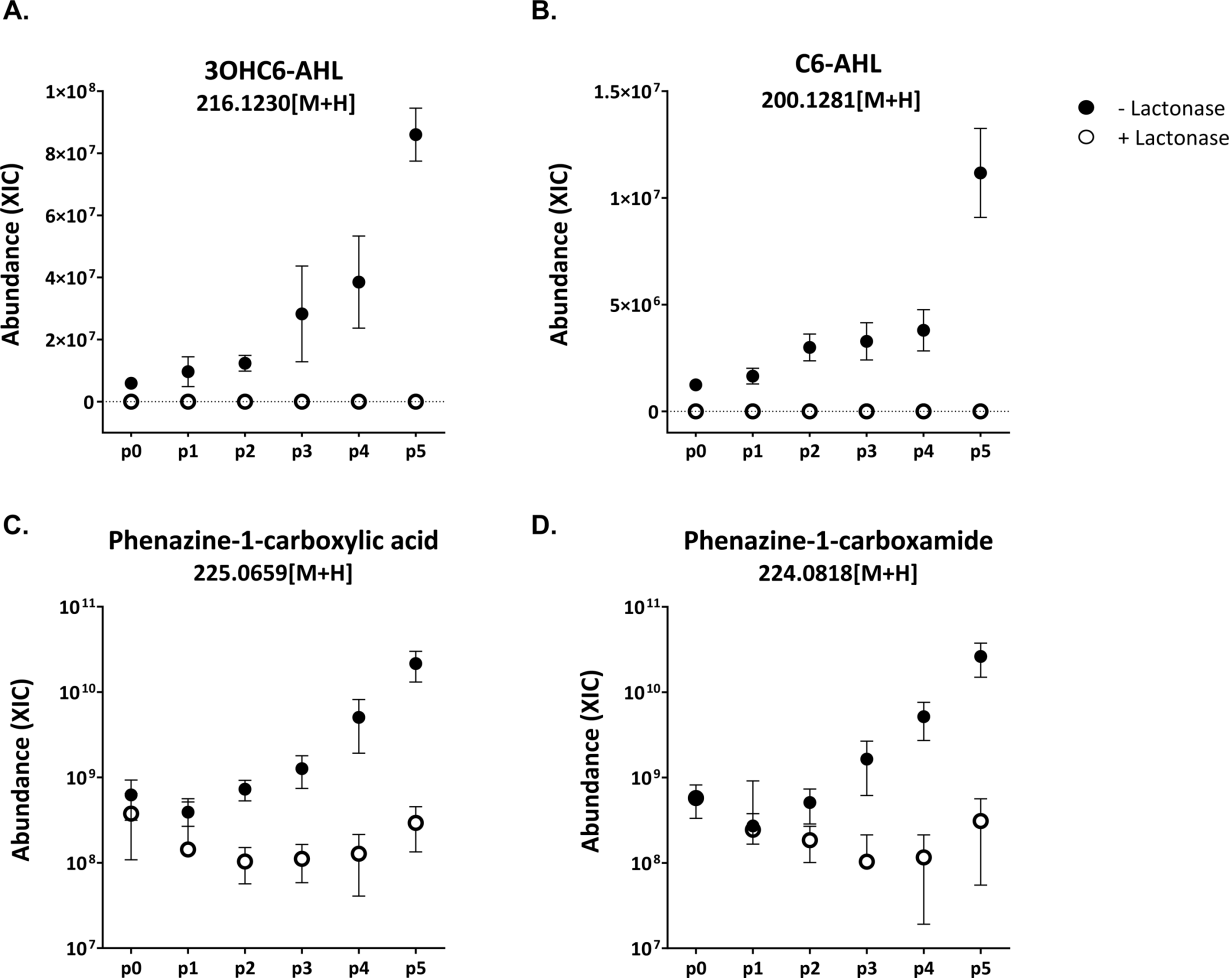
Relative abundance of secondary metabolites in lactonase-treated cultures (open circles) and untreated cultures (closed circles). Four relevant secondary metabolites were identified via mass spectrometry: 3OHC6-HSL (**A**), C6-HSL (**B**), phenazine-1-carboxamide (**C**), and phenazine-1-carboxylic acid (**D**). Each data point represents the average of three replicates. Error bars indicate the standard error of the mean.

In order to assess the relationship between community metabolic profiles and treatment, we used Pearson’s correlation analysis to compare metabolite abundances. Overall, 5,825 compounds were correlated with lactonase treatment (*r* = 0.5). Of those, 292 were significantly (*P* < 0.05) correlated with lactonase treatment (Table S7). Among the top differentially abundant compounds, there was a strong association between treatment and metabolite abundances for a diverse range of compounds, including both identified and unidentifiable metabolites (Fig. 3). Four biologically relevant metabolites differentially expressed between treatments were identified. Compounds 1,605 and 1,250 were identified as AHLs (3OHC6-HSL [*P* < 0.1] and C6-HSL [*P* < 0.05], respectively), and metabolites 1,814 and 1,833 were identified as phenazine derivatives (phenazine-1-carboxamide [(*P* < 0.001] and phenazine-1-carboxylic acid [*P* < 0.05]) (Fig. 4) known to be 3OHC6-HSL-controlled in other *Pseudomonas* strains (35–38). Although compound 1,605 did not meet the conventional significance threshold of *P* < 0.05, it exhibited a strong correlation with treatment and was among the top differentially abundant compounds (*r* = 0.63), thus making this compound biologically relevant and undoubtedly responsive to treatment given its putative identification as an acyl homoserine lactone. Furthermore, metabolite expression varied considerably across passages, with treatment effects becoming more pronounced over time (Fig. 3).

### AHLs and phenazines are decreased with the addition of lactonase

In cultures without lactonase addition, the relative abundance of AHLs (3OHC6-HSL and C6-HSL) increased steadily with each passage. By p5, the relative abundance of AHLs was nearly an order of magnitude greater compared to the previous passage. This is likely reflective of the positive feedback loop common in AHL-based QS signaling (39, 40). In lactonase-treated cultures, however, both signaling molecules were nearly undetectable compared to controls (Fig. 4A and B). Similarly, the relative abundance of phenazine compounds (phenazine-1-carboxamide [PCN] and phenazine-1-carboxylic acid [PCA]) increased across passages in untreated cultures. In lactonase-treated cultures, PCN abundance decreased significantly by p2 and remained nearly two orders of magnitude lower relative to PCN abundance in untreated cultures until the end of the experiment. Likewise, PCA abundance also showed a significant decrease in the presence of lactonase compared to the control (Fig. 4C and D).

### Lactonase disrupts antagonistic behavior between select microbes

**Fig 5:**
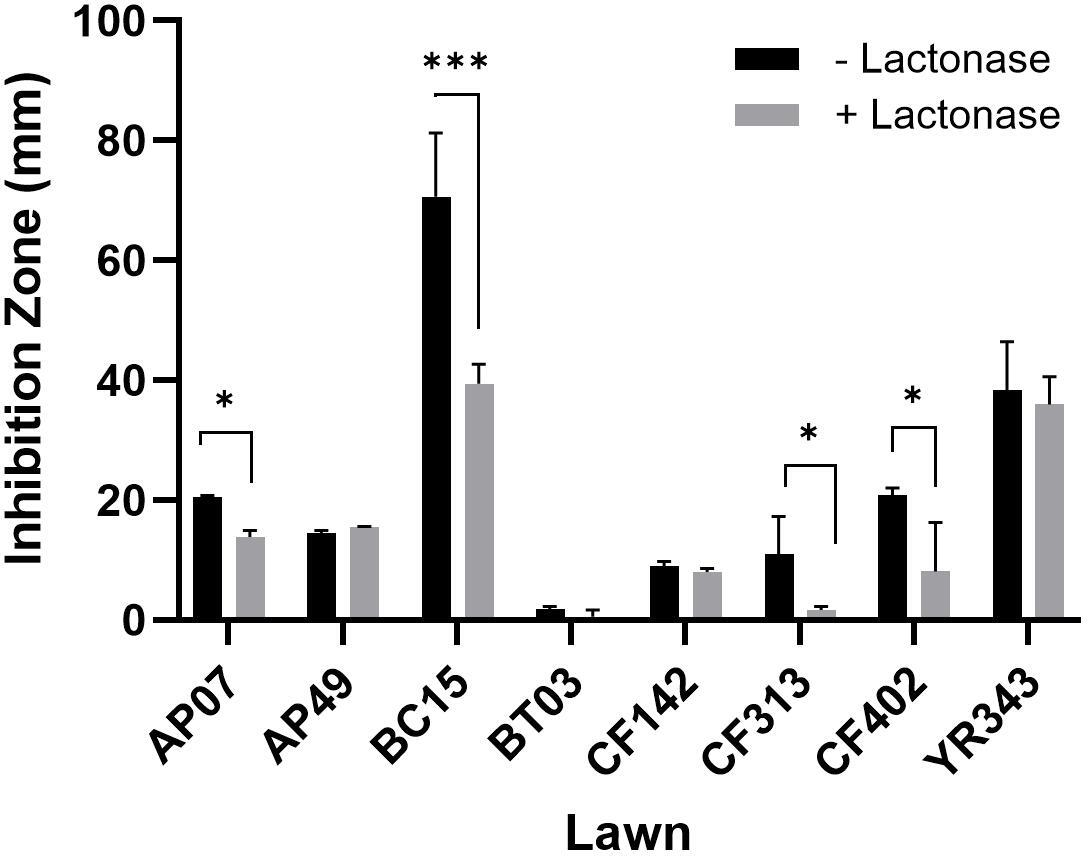
Impact of AiiA-lactonase on antagonistic interactions within the microbial community. The bar graphs represent the mean size of growth inhibitory zones produced by GM17 when overlaid with six lawn organisms (AP07, AP49, BC15, CF313, CF402, or YR343) in the presence (black bars) or absence (gray bars) of lactonase. Inhibition zones were quantified using the ObjectJ plugin in ImageJ. The error bars indicate the standard deviation (SD). Statistical significance was calculated using a two-way ANOVA (*P* < 0.01 [*] and *P* < 0.0001 [***]).

Because secondary metabolite production can be under QS control (41), we assessed whether lactonase could alter antagonistic behavior using a pairwise interaction assay (42). For each treatment, we prepared nine of the ten community members as lawn organisms: *Caulobacter* sp. AP07, *Sphingobium* sp. AP49, *Bacillus* sp. BC15, *Paraburkholderia* sp. BT03, *Rhizobium* sp. CF142, *Variovorax* sp. CF313, *Duganella* sp. CF402, *Pseudomonas* sp. GM17, and *Pantoea* sp. YR343. *Streptomyces* sp. YR139 was not utilized as a lawn due to the morphology of its colony-forming units on agar plates. All individual strains were spotted onto lawns and served as potential inhibitor organisms. Regardless of the inclusion of lactonase, inhibition was observed by a zone of clearing around GM17 on six of the nine lawns tested: AP07, AP49, BC15, CF313, CF402, and YR343 (Fig. S7). These results are consistent with previous findings in a rich medium where GM17 demonstrated antagonism against eight community members (24). When treated with lactonase, inhibition by GM17 decreased in AP07, BC15, CF313, CF402, and YR343 lawns by 32%, 44%, 84%, 61%, and 6%, respectively. This decrease was significant (*P* < 0.05) for all strains except YR343 (Fig. 5) and suggests that QS partially modulates antagonistic behavior by GM17.

## DISCUSSION

In bacterial communities, quorum sensing (QS) allows cells to respond to population density and AI concentration to regulate social responses, a crucial aspect of maintaining the balance and functionality of microbial communities. QS homologs are prevalent in natural microbial communities in terrestrial systems (15, 29, 31). In these environments, Proteobacteria are the dominant organisms known to produce AHL signaling molecules. AHL-mediated QS has been demonstrated to impact community composition and function of microbial constituents in controlled laboratory experiments (e.g., bioreactors and mesocosms) (43–46). Further, exogenous addition of AHLs altered community composition in wastewater treatments (43, 47), and addition of AHLs shifts the community composition of nitrifying and ammonium-oxidizing bacteria under multiple conditions (46). In contrast, AHL amendments to anaerobic ammonium-oxidizing communities instead altered community metabolism (45). Likewise, disrupting QS in a process called quorum quenching has been reported to impact microbial communities associated with *Citrus reticulata* (48), on immersed steel coupon surfaces (49), and in continuous bioreactor systems (50–52). These studies highlight the importance of QS in modulating the structure, function, and dynamics of microbial communities, supporting the idea that QS signaling contributes to shaping microbial community assembly and interactions within.

More recently, the use of SynComs has been employed to investigate the role of QS in microbial communities. For instance, AHL-based signal exchange between related species has been explored among *Vibrio* species (11, 14, 20). However, these studies are limited by only addressing QS-mediated signal exchange between related populations. Other investigations have focused on QS-mediated interactions between a pair of distantly related species (12, 15, 53–55), but these studies do not address complex interactions that occur in a multispecies consortium. In a recent study, QS knockouts in a dominant and antagonistic community member resulted in a more cooperative community without fully altering community composition (22). Another study using a semisynthetic community found that secondary metabolites altered community composition (21). Here, we assessed the effect of QQ on microbial community structure and dynamics in a ten-member SynCom using purified lactonase. To visualize shifts in community dynamics in response to QS disruption, we compared community composition and metabolite expression between lactonase-treated and untreated communities, as well as antagonistic behavior in pairwise interactions.

When comparing metabolites extracted from treated and untreated communities at 24 and 48 h, lactonase was able to disrupt AHL-based signaling in our polymicrobial community for 48 h. These results demonstrate that lactonase remained active during the growth period prior to each passaging, maintaining its ability to disrupt AHL-based communication. Suppression of AHLs by lactonase enabled us to explore the broader impacts of AHL disruption on community structure and dynamics.

Analysis of community composition data (16S rRNA amplicon) in the context of QQ revealed shifts in relative abundances rather than membership. The dominant strains (*Pseudomonas* sp. GM17, *Pantoea* sp. YR343, *Sphingobium* sp. AP49, and *Variovorax* sp. CF313) persisted in both lactonase-treated and untreated cultures; however, their relative abundances were markedly altered by the presence of lactonase. Most notably, the relative abundance of *Pseudomonas* sp. GM17 decreased significantly in lactonase-added cultures, while the relative abundance of YR343 increased. These findings are supported by those of previous investigations exploring the role of signaling in synthetic communities (21, 22). Our results indicate that the PD10 SynCom composition stabilizes in a similar manner regardless of QQ with lactonase. Only at later passages did treatment with lactonase drastically alter the relative abundance of GM17 and YR343. This delayed response to QQ is consistent with the observations of previous studies demonstrating that microbial communities may exhibit a degree of resilience to perturbations, with compositional shifts and community dynamics becoming apparent only after prolonged exposure to lactonase (52). Persistent community members, including AP49, CF142, and CF313, displayed similar relative abundances regardless of treatment, suggesting these strains may be impacted less by QQ.

Broader metabolic changes in both treated and untreated communities were evident in untargeted metabolomic profiles. Our results indicate that passage, treatment, and their interaction have a significant impact on community metabolomic profiles. Alterations in QS have been shown to induce global metabolic shifts in microbial communities (21, 45, 56). A major limitation to untargeted metabolomics is the large number of unidentified metabolites. Future efforts to identify the unknown metabolites differentially expressed between treatments and which metabolites are produced by individual strains may lead to the discovery of secondary signaling molecules or modulators of community dynamics. Despite this shortcoming, we identified two categories of secondary metabolites: AHL signaling molecules and phenazine derivatives in our metabolomics data. In general, lactonase-treated cultures produced less AHL signals and phenazine derivatives, phenazine-1-carboximide (PCN) and phenazine-1-carboxylic acid (PCA). It is generally accepted that AHLs mediate cooperative and competitive interactions by regulating metabolite exchange in the form of antimicrobial production and the synthesis of public goods, thereby shaping microbial communities (57). There is growing support that phenazines may act as redox-active metabolites (RAM) and impact microbial communities. For example, RAMs are posited to be keystone metabolites influencing microbial community dynamics by serving as signaling agents, supporting energy conservation, and facilitating nutrient acquisition (58). Among the most extensively studied examples of RAMs are phenazines, produced by various soil-dwelling species, such as Actinobacteria and Proteobacteria. Widely known for its bioactivity is PCN, an environmentally relevant phenazine derivative, which has been demonstrated to inhibit both bacterial (59, 60) and fungal pathogenic activity (61, 62). In addition, PCN is regulated by *lux* homologs, PhzR/PhzI, in several *Pseudomonas* strains (36, 37). Genomic analysis of GM17 reveals a QS system upstream of its phenazine biosynthetic gene cluster. This QS system is 99% identical to PhzR/I in *Pseudomonas chlororaphis* subsp. c*hlororaphis* DSM 50083, and produces 3OHC6-HSL (33), which in turn modulates the expression of phenazines (35–37, 63). Regulation of PCN production through QS systems highlights the complex interplay between environmental cues, metabolic responses, and community dynamics. The role of RAMs like PCN in shaping microbial interactions, particularly their dual function as both antimicrobials and signaling molecules, warrants exploration. Investigating how these metabolites influence not only microbial competition but also cooperation within communities could provide new insights into their ecological importance.

Coupling community composition data (16S rRNA amplicon) with metabolic profiles suggests that metabolic changes occur earlier, and potentially drive the observed shifts in community dynamics over time. This is further supported by pairwise interactions. For instance, AHL-inactivation leads to decreased inhibition of several community members by GM17 in pairwise interaction assays (Fig. 5). Furthermore, the observed decrease in the relative abundance of GM17, a strain known for its antagonistic behavior, aligns with previous findings that QQ can mitigate competitive interactions by impacting the production of antimicrobial compounds (24). While the exact mechanism of inhibition by GM17 is not yet defined, it is possible this antimicrobial activity is in part related to phenazine production, which is likely regulated by QS in GM17. Given that PCN exhibits antimicrobial properties in related species (59–62), it may be a contributing factor in the ability of GM17 to dominate by inhibiting competing strains.

The addition of lactonase disrupts both AHLs and phenazine derivatives in our community metabolic profiles. Further, the data show this disruption impacts antagonism by GM17 in pairwise interactions. However, the addition of lactonase does not completely abolish inhibition, suggesting that GM17 possesses other antimicrobial compounds or inhibitory mechanisms that are not QS-regulated. In support of this, a related *Pseudomonas* strain with deletions in its QS pathways exhibited inhibitory effects against competitors (36). It is evident that introducing lactonase disrupts this regulatory pathway, reducing antagonism and contributing to a fitness disadvantage for GM17, which ultimately results in its decreased abundance within the community. These observations are supported by several studies where a variety of lactonases have been demonstrated to decrease antagonistic behavior in *Pseudomonas* strains (64–70).

Microbial communities and factors that drive assembly represent a dynamic and evolving field of study. Early work in this field demonstrated pairwise interactions influence community composition (71). Other studies concluded environmental factors dictated community structure (72). A recent review discusses the influence of trophic interactions in the form of metabolic exchange as the central drivers of microbial community assembly (3). Here, we posit the differences between lactonase-treated and untreated constructed communities may be attributed to secondary succession dynamics. This ecological process occurs following a disturbance and results in a change of resources and densities of individuals in the community (73). The inhibition ecological model states that early colonizers dominate the community until a disturbance removes them, allowing for later successional species to replace them (74). Among our ten-member consortium, *Pseudomonas* sp. GM17 has been previously established as a dominant community member and has readily outcompeted community members regardless of the seeding density (24, 25). While lactonase impacts the entire community, our metabolic data suggest its effects are particularly pronounced on GM17. We observed high levels of 3OHC6-HSL, an AHL produced by *Pseudomonas* sp. GM17 (33), in untreated cultures. These levels were significantly reduced in lactonase-treated cultures. The disruption of QS in *Pseudomonas* sp. GM17 likely creates a fitness disadvantage, altering resource allocation and creating opportunities for less competitive community members to thrive. The observed shifts in community composition, with the emergence of previously less abundant species, further support this interpretation, suggesting a transition toward a new equilibrium state driven by secondary succession dynamics.

### Conclusions

The observations presented here provide insights into the role QS plays in microbial community structure. While disruption of QS can alter community structure, it does not impact community membership. The shift in community structure is likely a result of global changes in metabolites. These alterations in metabolites may facilitate decreased antagonistic behavior. Our results support that the establishment of stable microbial communities may protect against quorum quenching, which may otherwise destabilize natural communities. This conclusion may be particularly relevant in natural soil systems where quorum sensing could be impacted by both biotic and abiotic factors (i.e., enzymes such as lactonases and pH). To our knowledge, this study is the first to address the role of QS in a synthetic microbial community by enzymatically disrupting AHL-mediated quorum sensing, contributing to the growing knowledge base of cell-to-cell communication in microbial communities.

## MATERIALS AND METHODS

### Bacterial strains, media, and culturing

Ten representative bacterial strains, originally isolated from *Populus deltoides*, were selected to represent the *Populus* microbiome at the genus level (28). The ten-member community (PD10) includes *Caulobacter* sp. AP07, *Sphingobium* sp. AP49, *Bacillus* sp. BC15, *Paraburkholderia* sp. BT03, *Rhizobium* sp. CF142, *Variovorax* sp. CF313, *Duganella* sp. CF402, *Pseudomonas* sp. GM17, *Pantoea* sp. YR343, and *Streptomyces* sp. YR139 (26, 27, 29, 75). All strains were streaked from freezer stocks onto R2A agar (per liter: 3.12 g R2A, 15 g agar) and incubated at 25°C for at least 24 h. Except for CF142, all strains were then inoculated into 10 mL liquid MOPS minimal media with glucose (MOPS) (76) and incubated at 30°C, shaking (220 rpm) for 48 h. CF142 was inoculated in R2A liquid media and allowed to grow for 48 h. Following incubation, 10 mL of the CF142 culture was collected and washed to remove residual media. Washed cells were inoculated in 10 mL MOPS and sequentially used to set up serial passage experiments described below. Growth was monitored using OD_600_ (Genesys 20, Thermo Scientific Inc., Waltham, MA).

### Lactonase serial passage experiment

The AiiA-lactonase enzyme was purified from recombinant *Escherichia coli* as an MalE maltose binding protein fusion, as described previously (77). Two independent serial passage experiments were performed as follows: individual bacterial cultures were grown as described above and then diluted to the OD_600_ value of the least turbid member (BC15 OD_600_ = 0.14). Diluted cultures were mixed in equal volumes in a 250 mL flask. One milliliter from the combined culture (PD10) was transferred to 9 mL MOPS in triplicate for each treatment, with and without lactonase. Purified MBP-lactonase (10 µg/mL) was added to one set of triplicate cultures (32, 78, 79). All cultures were incubated at 30°C, shaking (220 rpm). Every 48 h, 1 mL of PD10 was transferred to 9 mL fresh MOPS. Lactonase was added at each passage for treated cultures. Lactonase-free cultures served as a control for all experiments. Cultures were serially passaged in this manner for a total of five passages, as described previously (24). At each time point (initial [t0], passage 0 [p0], passage 1 [p1], passage 2 [p2], passage 3 [p3], passage 4 [p4], and passage 5 [p5]), biomass (1 mL) was collected for dry cell weight and DNA extracted for 16S rRNA amplicon sequencing. Additionally, the culture supernatant was harvested for metabolite extraction.

### 16S amplicon sequencing

DNA was extracted from harvested cell pellets (1 mL) using the Qiagen DNeasy Blood and Tissue Kit (Qiagen, Valencia, CA) following the manufacturer’s protocols. Following extraction, DNA was quantified using a Qubit fluorometer (Invitrogen, Thermo Fisher Scientific, Waltham, MA) and diluted to >100 ng/mL. Diluted DNA was prepared for sequencing using the Quick 16S Plus NGS One-Step Library Prep Kit (Zymo Research Corp., Irvine, CA) and pooled per the manufacturer’s protocol. Pooled libraries were denatured with 0.2 N sodium hydroxide and then diluted to the final sequencing concentration. Due to low base diversity of the 16 S rRNA amplicon products, PhiX control DNA was added to increase overall base diversity for the sequencing runs. Libraries were loaded into the sequencing cassette (v2 chemistry), and a paired end (2 × 251-cycle) run was completed on an Illumina MiSeq Instrument (Illumina, San Diego, CA). Included in each sequence run were ZymoBIOMICS microbial community DNA standards and nuclease-free water (Zymo Research Corp., Irvine, CA). Demultiplexed reverse reads with quality were imported into qiime2 using the CasavaOneEightSingleLanePerSampleDirFmt function (version 2024.5) (80). Primers (341 f (CCTACGGGDGGCWGCAG, CCTAYGGGGYGCWGCAG, 17 bp) and 806 r (GACTACNVGGGTMTCTAATCC, 24 bp) were trimmed using Cutadapt (v4.9) (80, 81). Downstream analysis was based off of previous work (24).

### Metabolite extraction and analysis

We removed cells from cultures (7 mL, at each passage) by centrifugation (45 min at 2,125 × *g*) and extracted metabolites as previously described (79). AHL presence in an aliquot of culture extracts was assayed using the relaxed-specificity reporter *Agrobacterium tumefaciens* KYC55 (pJZ372, pJZ384, and pJZ410) (32, 78, 79). The remaining sample, along with blank media controls, was dried and stored at 4°C for metabolomic analysis. Samples were then resuspended in an organic solvent (70% acetonitrile and 0.1% formic acid) prior to ultra-high performance liquid chromatography tandem mass spectrometry (UHPLC-MS/MS) measurements, which were obtained using a ThermoFisher Vanquish coupled to a ThermoFisher Q-Exactive Plus mass spectrometer, as previously described (82). Briefly, for each sample, 10 µL was injected and allowed to flow across an in-house-constructed nanospray analytical column (75 µm × 150 mm) packed with a 1.7 µm C18 Kinetex RP C18 resin (Phenomenex). Chromatographic separation was achieved across a 30 min linear gradient (250 nL/min flow) from 5 to 100% organic solvent (70% acetonitrile and 0.1% formic acid). Full-scan MS spectra were collected from 135 to 2,000 *m/z* at 70,000 resolution, with a top-N method (*N* = 5) data-dependent acquisition strategy. Fragmentation of precursor ions used stepped collision energies of 10, 20, and 40 eV, with a dynamic exclusion of 10 s to avoid oversampling abundant metabolites. Spectral matching and quantification analyses were performed using Thermo Scientific Compound Discoverer (CD) v3.3.1 using default settings. The node “mzVault” was selected for spectral matching against the high-resolution NIST 2020 spectral library and the GNPS public spectral library (downloaded December 2023). Compounds with MS/MS fragment matching score of >0.5 support a Class 2 identification (83). Features with poor peak integration were resolved through automated data processing, where peak areas from indistinguishable features (based on annotated molecular weight and delta mass) were aggregated using a custom R script (available at https://github.com/dlcarper/Metabolite-Condensing-Script). Relative abundances of each compound were assessed by the integrated peak heights across samples. Skyline v22.2 (84) was used for manual curation of metabolite spectral matching and the relative abundances measured in this study.

### Pairwise interactions

Pairwise interactions were assessed using a modified Burkholder plate-based assay (mBA) (42). Briefly, 48 h cultures were diluted 1:10 in fresh MOPS. Cultures below an OD_600_ of 0.3 were left undiluted. Lawn organisms (AP07, AP49, BC15, BT03, CF142, CF313, CF402, GM17, and YR343) were inoculated in molten MOPS agar (~45°C) at 1:1,000 concentration (50 µL into 50 mL). A concentration of 10 µg/mL of MBP-lactonase was included for lactonase-treated lawns. The molten agar was gently mixed and poured into a square Petri plate. Lawns were prepared in duplicate. Once agar solidified, inhibitor organisms were spotted (5 µL) on top and allowed to dry. Plates were incubated at 25°C, and results were recorded using an Epson (Perfection V850) scanner after approximately 4 days of growth, utilizing the transparency unit feature to capture transmitted light to increase distinct edges and saved as a JPG image with minimal compression at 600 DPI.

### Statistical analysis and visualization

For display of normalized 16S rRNA amplicon sequencing data, stacked bar plots were generated in GraphPad Prism. To assess community composition differences, Bray-Curtis dissimilarity was calculated on normalized 16S rRNA amplicon sequencing data. To assess statistical differences in multivariate data, we used a permutational multivariate analysis of variance (PERMANOVA) and subsequently plotted on a principal coordinates analysis (PCoA) in R (v4.4.1 R Core Team 2021). Pairwise *t*-tests were performed to assess differences in the relative abundance of individual community members at each time point using GraphPad Prism. Biologically relevant metabolite identifications from Compound Discoverer (v3.3.1) were visualized in GraphPad Prism. Pearson’s correlation was conducted to determine metabolic changes associated with treatment and passage number. An analysis of variance (ANOVA) was used to identify metabolites with significant differences across treatment groups. Overall metabolite differences were determined by Bray-Curtis distance, visualized by non-metric multidimensional scaling (NMDS), and statistical significance determined by PERMANOVA. Pairwise interaction assays were quantified with ObjectJ (85, 86) to measure zones of inhibition in ImageJ (87). The resulting data were imported, visualized, and analyzed in R (v4.4.1 R Core Team 2021). ANOVA was conducted using the aov function (stats package, v4.4.1 R Core Team 2021).

## Data Availability

All raw and processed sequence data were deposited in the NCBI Sequence Read Archive (SRA) under BioProject ID PRJNA1190957. Metabolomics data are deposited in the MassIVE data repository, accession number MSV000096353.

## SUPPLEMENTAL MATERIAL

Supplemental Figures msphere.01050-24-s0001.docx Figures S1 to S7.

Supplemental Tables msphere.01050-24-s0002.xlsx Tables S1 to S7.

ASM does not own the copyrights to Supplemental Material that may be linked to, or accessed through, an article. The authors have granted ASM a non-exclusive, world-wide license to publish the Supplemental Material files. Please contact the corresponding author directly for reuse.
