# Supplementary material for: Quorum sensing modulates microbial community structure through regulation of secondary metabolites
Source: mSphere. 2025 Jun 20;10(7):e01050-24. doi: 10.1128/msphere.01050-24 (PMC12306166; doi:10.1128/msphere.01050-24)
Supplement: Supplemental Figures — Figures S1 to S7. [file msphere.01050-24-s0001.docx]

Supplementary Information

For

Quorum sensing modulates microbial community structure through regulation of secondary metabolites

**April Armes^a^**, Amy L. Schaefer^b^, Leah H. Hochanadel^a^, Dawn M. Klingeman^a^, Dana L. Carper^a^, Paul E. Abraham^a^, Larry M. York^a^, Alyssa A. Carrell^a^, Mitchel J. Doktycz^a#^ and Dale A. Pelletier^a#^

^a^ Biosciences Division, Oak Ridge National Laboratory, Oak Ridge, Tennessee 37831, United States.

^b^ University of Washington, Seattle, WA

**Supplemental Figure 1.** AHL activity with (black bars) and without (grey bars) lactonase at 24 and 48 h for PD10 communities in MOPS + glucose at 0.1X concentration. Each data point is the average of three replicates. Baseline for blank media control is indicated by red dotted line. Statistical significance was calculated using a two-way ANOVA [p < 0.001 (**), p < 0.0001 (****)].


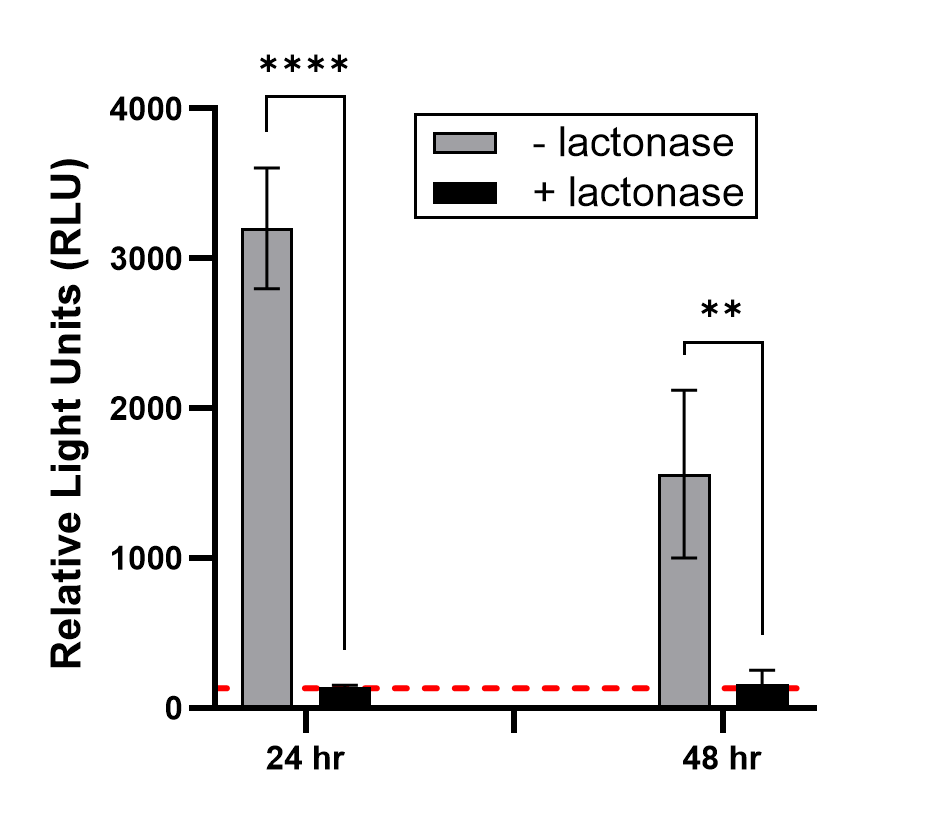


**Supplemental Figure 2.** Growth parameters of PD10 communities as a function of optical density (A) and dry cell weight (B) in the absence [circles (A), grey bars (B)] and presence [triangles (A), black bars (B)] of lactonase. A total of six replicates from two independent experiments are plotted. Statistical analysis was calculated using a two-way ANOVA. No statistical differences were found between treatment or passage.

**
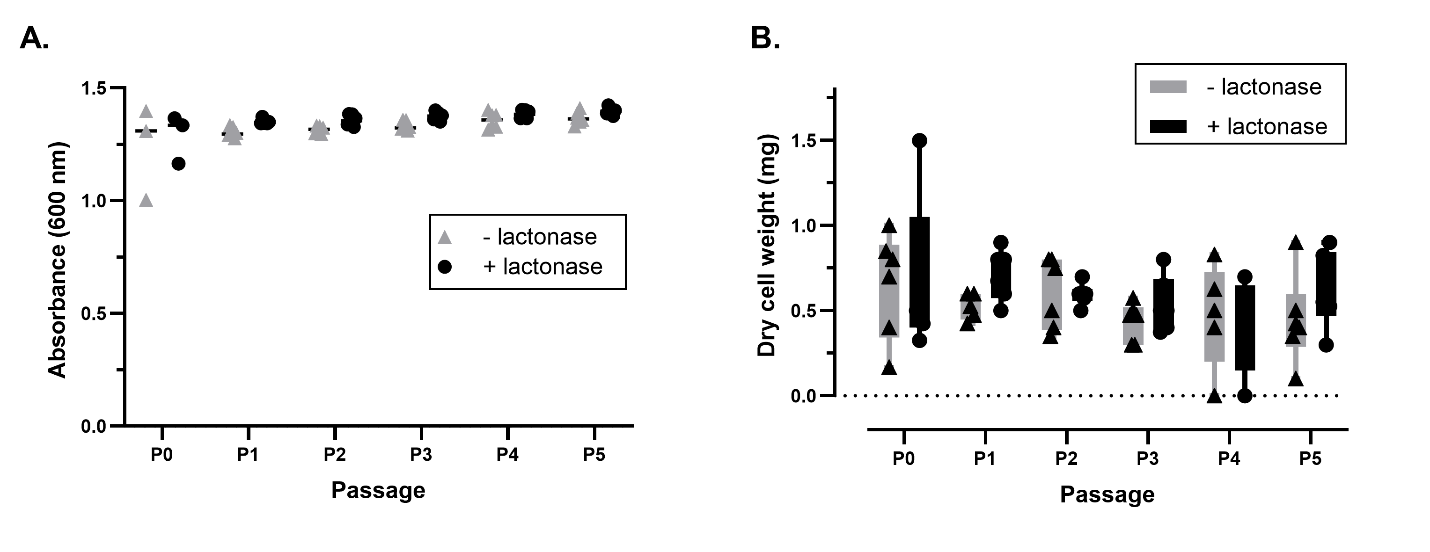
**

**Supplemental Figure 3.** Relative abundance (16S rRNA amplicons) of top four taxa at passage 5 treated with (black boxes) and without (grey boxes). Box and whisker plots show the minima and maxima of each data set. Statistical significance was calculated using Welch’s t-test and corrected for **
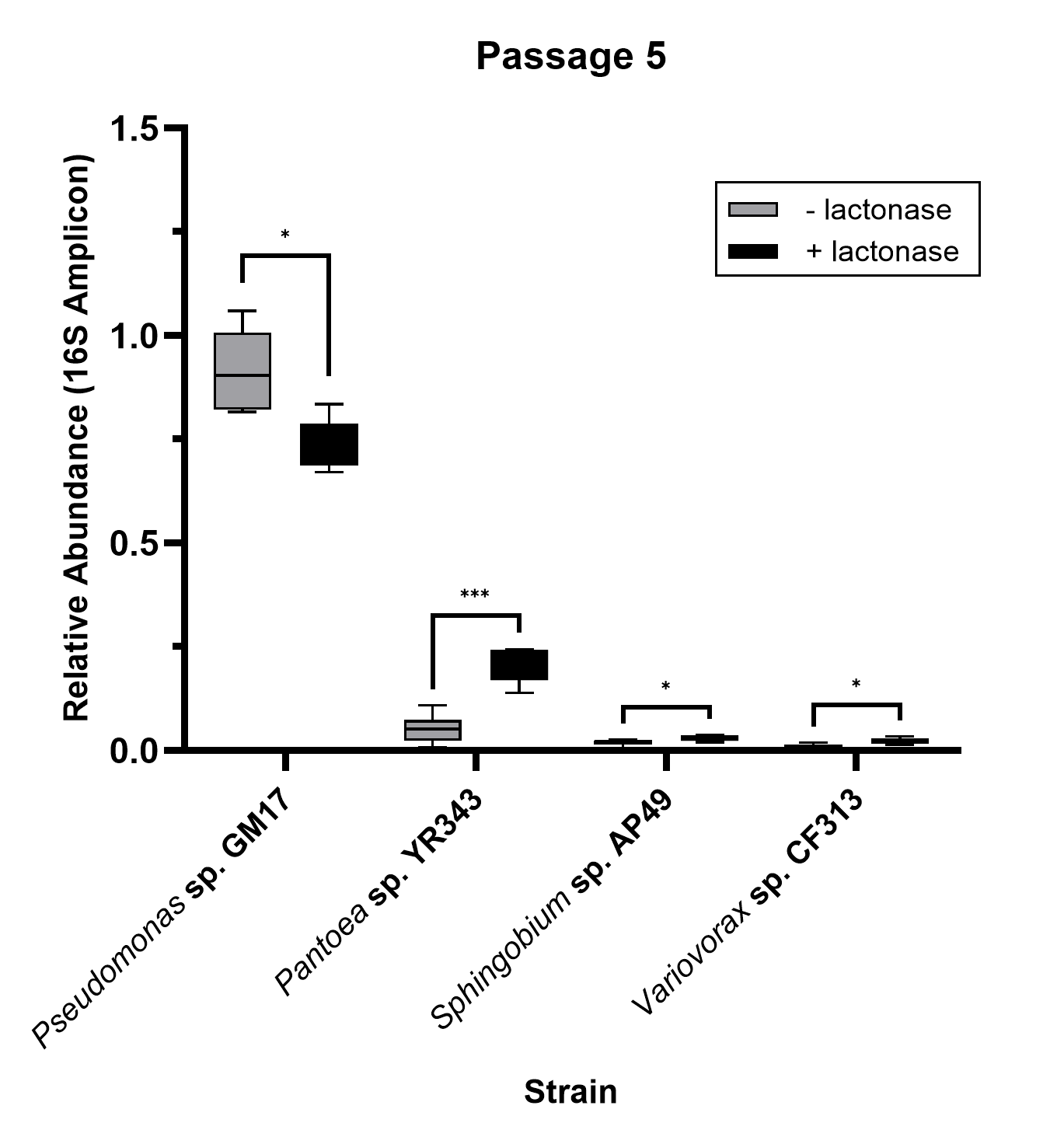
**multiple comparisons using Holm-Sidak method [p < 0.001 (***), p < 0.01 (*)].

**Supplemental Figure 4.** Principal Coordinates Analysis (PCoA) of beta diversity analysis using Bray-Curtis dissimilarity for all synthetic communities at each passage based on 16S rRNA amplicon sequences. Each data point represents one culture sample. Passage is denoted by color and treatment is designated by shapes with and without lactonase as circles and triangles, respectively. PCo1 represents the dissimilarity due to passage, while PCo2 indicates the dissimilarities due to treatment, with percent variance indicated in parentheticals.


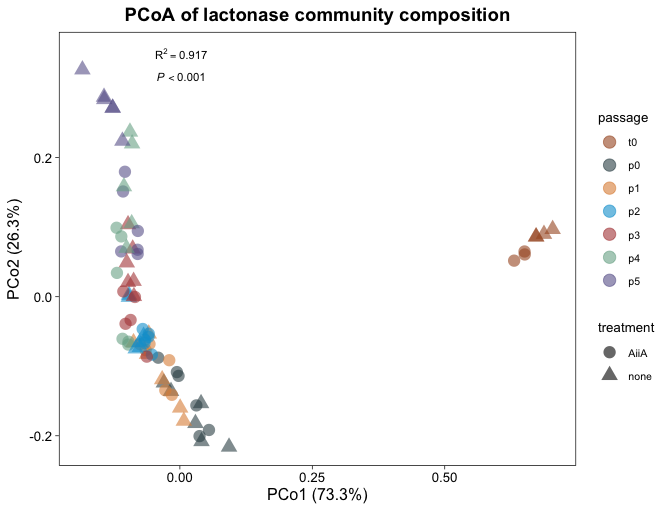


**Supplemental Figure 5.** Hierarchical clustering of metabolomic data distinguished treatment groups based on their metabolite profiles. (AKA All changing metabolites averaged)


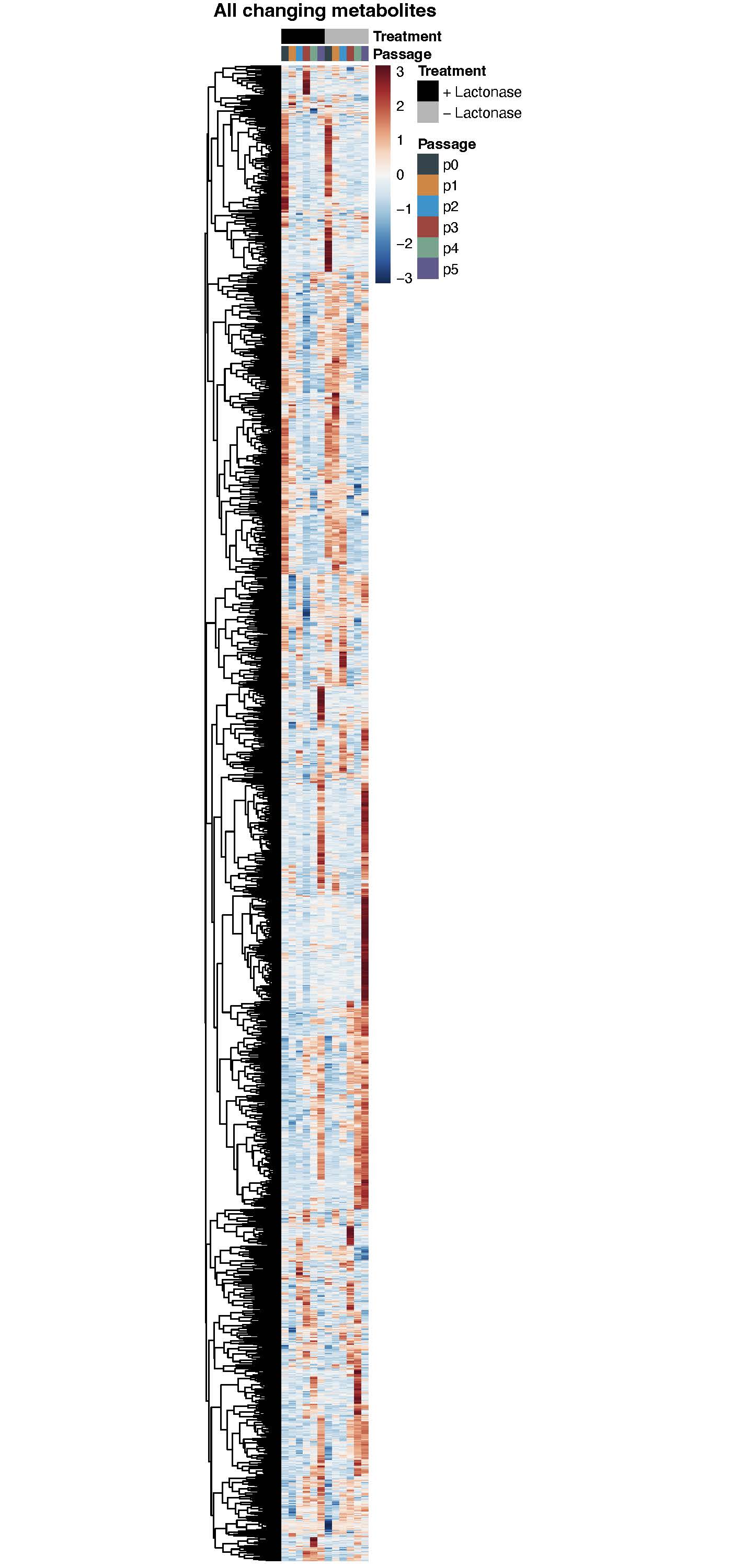


**Supplemental Figure 6.** Unconstrained Non-metric Multidimensional Scaling (NMDS) of PD10 community metabolites for each passage. Each data point represents one sample culture. Passage is denoted by color and treatment is designated by shapes with and without lactonase as circles and triangles, respectively. NMDS axes represent a two-dimensional ordination of the samples based on their pairwise dissimilarities, with the relative distances between points reflecting the similarity of microbial community metabolic profiles. Numbers on both axes indicate stress value denoting goodness of fit, with values ≤ ±0.2 considered a good representation of the original data.

−0.02

0.00

0.02

−0.02

0.00

0.02

NMDS1

NMDS2

Passage

p0

p1

p2

p3

p4

p5

Treatment

Lactonase

+

−

Lactonase

**Supplemental Figure 7. Images of *Pseudomonas* sp. GM17 spotted (5 µL) on top of lawns:** *Caulobacter* sp. AP07 (A), *Sphingobium* sp. AP49 (B) , *Bacillus* sp. BC15 (C), *Paraburkholderia* sp. BT03 (D), *Rhizobium* sp. CF142 (E), *Variovorax* sp. CF313 (F), *Duganella* sp. CF402 (G), and *Pantoea* sp. YR343 (H). Zones of clearing surrounding GM17 spots indicate inhibition.

**
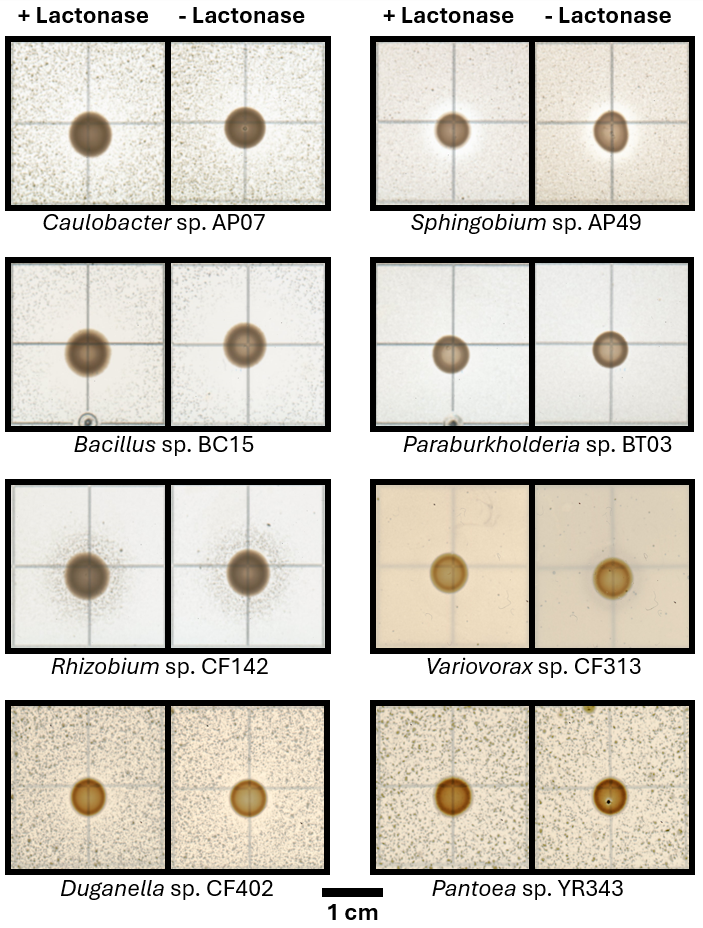
**
